# Hypothalamus and amyotrophic lateral sclerosis: potential implications in sleep disorders

**DOI:** 10.3389/fnagi.2023.1193483

**Published:** 2023-07-03

**Authors:** Valentina Gnoni, Stefano Zoccolella, Alessia Giugno, Daniele Urso, Ludovica Tamburrino, Marco Filardi, Giancarlo Logroscino

**Affiliations:** ^1^Center for Neurodegenerative Diseases and the Aging Brain, University of Bari Aldo Moro at Pia Fondazione “Card. G. Panico,” Tricase, Italy; ^2^Department of Neurosciences, King’s College London, Institute of Psychiatry, Psychology and Neuroscience, De Crespigny Park, London, United Kingdom; ^3^Neurology Unit, San Paolo Hospital, Azienda Sanitaria Locale (ASL) Bari, Bari, Italy; ^4^Department of Translational Biomedicine and Neurosciences (DiBraiN), University of Bari Aldo Moro, Bari, Italy

**Keywords:** amyotrophic lateral sclerosis, hypothalamus, sleep disorders, circadian rhythm, neurodegeneration

## Abstract

Amyotrophic lateral sclerosis (ALS) is a devastating neurodegenerative disease that affects both motor and non-motor functions, including sleep regulation. Emerging evidence suggests that the hypothalamus, a brain region that plays a critical role in sleep-wake regulation, may be involved in the pathogenesis of ALS-related sleep disturbances. In this review, we have summarized results of studies on sleep disorders in ALS published between 2000 and 2023. Thereafter, we examined possible mechanisms by which hypothalamic dysfunctions may contribute to ALS-related sleep disturbances. Achieving a deeper understanding of the relationship between hypothalamic dysfunction and sleep disturbances in ALS can help improve the overall management of ALS and reduce the burden on patients and their families.

## Introduction

Amyotrophic lateral sclerosis (ALS) is a fatal neurodegenerative disease characterized by the progressive degeneration of upper (UMN) and lower motor neurons (LMN) (Kiernan et al., 2011).

Amyotrophic lateral sclerosis is traditionally considered to selectively present with motor symptoms. However, growing evidence has shown that patients experience a spectrum of non-motor symptoms, ranging from cognitive to autonomic, metabolic, and endocrine dysfunctions (Günther et al., 2016; Urso et al., 2022). ALS is indeed currently considered a multiple-system disease (Ludolph, 2017; Verde et al., 2017; Chiò et al., 2021; Logroscino et al., 2022). Among non-motor symptoms, sleep disorders and metabolic alterations (weight loss and hypermetabolism) are highly prevalent and negatively impact the prognosis and patients’ quality of life (Beswick et al., 2022). The hypothalamus is a central structure of the brain that represents a critical hub between central and peripheral signals and has a major role in the regulation of sleep/wakefulness and endocrine system (Berthoud, 2002; Morton et al., 2014; Hiller and Ishii, 2018). Hypothalamic alterations have been documented in several neurodegenerative disorders, including ALS (Vercruysse et al., 2018). Nonetheless, the relationship between hypothalamic dysfunction and sleep disorders in ALS has yet to be elucidated. This review aims to summarize studies on sleep disorders in ALS and discuss the potential role of hypothalamic dysfunction in sleep disorders in ALS.

## Materials and methods

A search of electronic databases to identify studies published in peer-reviewed journals starting from 1st January 2000 to 1st January 2023 was conducted. The following databases have been used to search for relevant keywords: PubMed, Web of Science and Scopus. Search terms used were *amyotrophic lateral sclerosis*, *sleep*, *sleep disorders*, *rest-activity rhythm*, *insomnia*, *parasomnia, excessive daytime sleepiness, periodic leg movements* and *rapid eye movement sleep behavior disorder.* As studies on sleep-related breathing disorders in ALS have been recently reviewed (Boentert, 2019), we decided to not systematically review these studies in the present review. Twenty-two studies fulfilled the criteria for full-text review, detailed information for each study is reported in Table 1.

**TABLE 1:** Summary of studies included within the review.

References	Sample	Mean age	Diagnosis according to	Clinical and instrumental assessment	Sleep assessment
Arnulf et al., 2000	13 ALS with diaphragmatic dysfunction 8 ALS without diaphragmatic dysfunction	60 ± 12 56 ± 9	El Escorial WFN (definite or probable)	Limb and Bulbar functional testing Manual muscle testing Spirometry Diaphragm electromyogram	PSG ESS
Atalaia et al., 2007	11 ALS with normal respiratory tests, no sign of diaphragm denervation and abnormal NPO	30 ± 77	El Escorial	Respiratory function tests Percutaneous oximetry Neurophysiological assessment of phrenic nerve and diaphragm	V-PSG
Lo Coco et al., 2006	14 ALS slow progression 10 ALS intermediate progression 14 ALS rapid progression	60.8 ± 13.3 60.3 ± 13.6 58.4 ± 9.5	El Escorial WFN revised	ALSFRS Appel ALS rating scale FVC	Nocturnal oximetry (every 4 months)
Diaz-Abad et al., 2018	43 ALS 43 controls	63.8 ± 11.5 61.3 ± 8.7	El Escorial WFN revised	ALSFRS revised BDI	PSQI ESS
Panda et al., 2018	40 ALS 190 controls	58.5 (44–75) Age-and sex matched to ALS	El Escorial	ALSFRS HADS	ESS PSQI *Ad hoc* questionnaire (based on case Western health reserve and sleep disorders questionnaire) IRLSRS
Devenney et al., 2021	64 ALS without cognitive and behavioral impairment 23 ALS with cognitive impairment 16 ALS with behavioral impairment 12 ALS with cognitive and behavioral impairment 98 bvFTD 37 ALS-FTD	61.54 ± 9.91 59.83 ± 9.71 60.81 ± 9.53 66.75 ± 11.55 62.98 ± 8.66 63.84 ± 8.66	El Escorial revised, Awaji criteria, gold coast criteria	King’s staging ACE-III	CBI revised
Gabery et al., 2021	9 ALS 8 healthy controls	N/A N/A	El Escorial revised (possible, probable, or definite)	ALSFRS revised ACE–III	CBI
Sun et al., 2021	204 ALS 206 healthy controls	53.5 ± 9.9 53.7 ± 12.7	Awaji criteria (definite or probable)	ALSFRS revised MMSE MoCA FAB HDRS HARS	PSQI ESS RBDSQ Clinical interview to assess RLS
Wei et al., 2021	224 ALS (King’s Stage 1) 193 ALS (King’s Stage 2) 86 ALS (King’s Stage 3) 44 ALS (King’s Stage 4)	54.0 ± 11.0 55.1 ± 11.8 55.1 ± 12.7 55.5 ± 9.5	El Escorial revised (possible, probable, or definite)	ALSFRS King’s staging ACE revised FAB HDRS HARS EuroQol five-dimensions questionnaire	ESS PSQI RBDSQ
Liu et al., 2018	121 ALS 121 healthy controls	53.01 ± 9.51 52. 3 ± 10.7	Awaji criteria (definite or probable)	ALSFRS revised MMSE MoCA FAB NPI FBI HDRS HARS	PSQI RBDSQ ESS Clinical interview to assess RLS PSG
Lo Coco and La Bella, 2012	43 ALS with fatigue 43 ALS without fatigue	62.6 ± 8.66 59.77 ± 11.47	El Escorial revised (Probable or Definite)	ALSFRS-R BDI FSS	PSQI ESS
Colville et al., 2007	26 ALS	64.12 ± 10.6	El Escorial revised (suspected, possible, probable, or definite)	ALSFRS revised	ESS NPO
Lo Coco et al., 2011	100 ALS 100 controls	59.9 ± 12 57.9 ± 12.8	El-Escorial WFN revised	ALSFRS revised BDI	PSQI Clinical interview to assess RLS ESS PSG (12 out of 100 ALS)
Desai, 2014	16 ALS with EDS 7 ALS without EDS	N/A	N/A	N/A	ESS PSG
Ramirez et al., 2008	60 ALS 60 controls	56.08 ± 12.26 N/A	El Escorial	ALSFRS FSS McGill Quality of Life Questionnaire Dyspnea analogical scale BDI	ESS
Choquer et al., 2021	27 ALS	66 ± 12	El Escorial revised	ALSFRS revised BDI	ESS PSQI PSG
Congiu et al., 2019	31 ALS 26 healthy controls	63.94 ± 10.17 62.19 ± 13.93	El Escorial revised	ALSFRS revised ALSSS	ESS PSQI BQ Restless legs syndrome diagnostic Interview IRLSRS RBDSQ V-PSG
Puligheddu et al., 2016	29 ALS 28 controls	63.6 ± 11.61 63.8 ± 12.19	El Escorial (possible or probable)	ALSFRS ALSSS	V-PSG
Lo Coco et al., 2017	41 ALS 26 healthy controls	60 (55–72) 60 (54–70)	El-Escorial WFN (definite or probable)	ALSFRS revised	Clinical interview to assess insomnia, RLS and RBD PSQI ESS V-PSG
Malekshahi et al., 2019	8 completely locked-in ALS	45.13 ± 20.82	N/A	N/A	Continuous PSG (48-h)
Limousin et al., 2011	13 ALS with RLS 56 ALS without RLS	72.62 ± 6.29 68.59 ± 10.27	El-Escorial revised (definite or probable)	ALSFRS	ESS Clinical interview to assess insomnia and RLS
Moszczynski et al., 2012	35 ALS 35 controls	64.03 ± 12.70 61.83 ± 12.66	N/A	ALSFRS revised Spirometry	ESS RLS questionnaire Periodic Limb movement questionnaire PSG

ALS, amyotrophic lateral sclerosis; WFN, world federation of neurology; PSG, polysomnography; ESS, Epworth sleepiness scale; NPO, nocturnal pulse oximetry; V-PSG, video-polysomnography; ALSFRS, ALS-functional rating scale; FVC, forced vital capacity; BDI, beck depression inventory; PSQI, Pittsburgh sleep quality index; HADS, hospital anxiety and depression scale; ACE-III, Addenbrooke’s cognitive examination III; CBI, Cambridge behavioral inventory; MMSE, Mini-Mental state examination; MoCA, Montreal cognitive assessment; FAB, frontal assessment battery; HDRS, Hamilton depression rating scale; HARS, Hamilton anxiety rating scale; RBDSQ, REM sleep behavior disorder screening questionnaire; RLS, restless leg syndrome; FAB, frontal assessment battery; NPI, neuropsychiatric inventory; FSS, fatigue severity scale; ALSSS, ALS severity scale; IRLSRS, restless legs syndrome severity rating scale; RBD, REM sleep behavior disorder.

## Sleep disorders in ALS

Sleep-related breathing disorders are the most common sleep disorders experienced by patients with ALS and have been extensively documented (Boentert, 2019). Changes in breathing during sleep may precede wake respiratory symptoms and occur in patients with normal respiratory function. However, as the disease evolves, the progressive respiratory and upper airway muscles weakness, as well as the diaphragmatic weakness, lead to nocturnal hypoxia and hypoventilation (Ahmed et al., 2016; D’Cruz et al., 2018). In this regard, Arnulf et al. (2000) showed that patients with diaphragmatic dysfunction had reduced or absent REM sleep, which might represent a possible protective mechanism against hypoventilation. Conversely, Atalaia et al. (2007) showed that sleep-related breathing disorders and reduction/absence of REM sleep may be present also in patients with unimpaired diaphragmatic function, which suggest a central drive dysfunction in ALS. Moreover, a longitudinal study has shown that over a 26-months follow-up period, half of the patients in the first 12 months and 70% of patients over the entire follow-up, develop chronic hypoventilation (Lo Coco et al., 2006).

Sleep disorders other than sleep-related breathing disorders have been less studied in ALS.

The majority of studies assessed sleep disturbances through self-report measures, namely the Pittsburgh Sleep Quality Index (PSQI), Epworth Sleepiness Scale (ESS) and questionnaire on quality of life. This trend has been highlighted in a recent review of ALS clinical trials which showed that sleep disturbances were assessed in only 12 studies (among 237) and exclusively through self-report measures (Beswick et al., 2022).

The study by Diaz-Abad et al. (2018) used the PSQI to assess habitual sleep quality and duration in ALS. The authors showed that 63% of ALS patients report poor sleep quality (i.e., PSQI > 5), compared to 37% of controls and that sleep quality is associated with the severity of depressive symptoms (Diaz-Abad et al., 2018). Panda et al. (2018) showed that sleep quality in ALS patients is significantly impaired in all components of PSQI and that 50% of patients report poor sleep quality. Similar results emerged in the study by Devenney et al. (2021) which used the Cambridge behavioral inventory (CBI). The authors reported a high prevalence of sleep disturbances across all ALS phenotypes. Notably, sleep disturbances were present in 99% of ALS patients with both cognitive and behavioral impairment, 80% of patients with exclusively behavioral impairment, 69.5% of patients with exclusively cognitive impairment and 70% of patients without cognitive or behavioral impairment.

However, in a subsequent study, Gabery et al. (2021) found no difference between ALS patients and controls in sleep disturbances assessed through the CBI. Sun et al. (2021) evaluated sleep quality in genetic and sporadic ALS. Genetic ALS patients presented higher PSQI score than sporadic patients with both groups presenting higher scores than controls. Finally, Wei et al. (2021) evaluated the influence of sleep quality on patients’ quality of life. The authors reported a high prevalence of poor sleep quality (57.19%) and showed that ALS patients with poor sleep quality had lower healthy utility scores.

Excessive daytime sleepiness is also highly prevalent in patients with ALS.

Liu et al. (2018) showed that excessive daytime sleepiness (EDS) was significantly more frequent in patients with ALS than in controls (26.4 vs. 8.3%). Patients with ALS with EDS had higher ALSFRS-R global scores and lower global score and delayed memory score at MMSE and MoCA than patients with ALS without EDS (Liu et al., 2018). Similarly, Lo Coco and La Bella (2012) reported that 24.2% of ALS patients present EDS. Colville et al. (2007) showed that 42% of patients with ALS report EDS although only 7% report severe sleepiness.

Conversely, the study by Diaz-Abad et al. showed that although ALS patients had higher ESS score than controls, only 14% of patients present EDS (ESS > 9) (Diaz-Abad et al., 2018). Noteworthy, ESS was not associated with sleep quality but with respiratory muscle weakness. On the contrary, in the study by Lo Coco et al. (2011) ALS patients who reported poor sleep quality showed higher ESS score. Desai (2014) showed that daytime sleepiness was not related to the degree of AHI or sleep-disordered breathing and hypothesized a dysfunction of central mechanisms. Sun et al. (2021) showed that EDS is more prevalent in genetic than in sporadic ALS and that both patient groups have more severe sleepiness than controls. Finally, two studies did not find any difference in daytime sleepiness between ALS patients and controls (Ramirez et al., 2008; Panda et al., 2018). Concerning primary sleep disorders, insomnia has been the most extensively studied and is a frequent complaint of patients with ALS (Lo Coco and La Bella, 2012; Boentert, 2019). The prevalence of insomnia in ALS may range depending on the study population and the subtypes of insomnia.

Lo Coco et al. (2011) showed that 48% of patients report middle and terminal insomnia and 32% initial insomnia, while general non-restorative sleep symptoms were reported by 29% of ALS patients. Panda et al. (2018) showed that insomnia is reported by 65% of ALS patients, specifically, 61% present difficulties initiating sleep, 88.5% difficulties in maintaining sleep, and 38.5% early morning awakenings. Finally, Choquer et al. (2021) showed that 69% of the ASL patients report insomnia, 55% of which report poor sleep quality suggesting a strong association between insomnia and poor sleep quality and that insomnia may be independent from respiratory disorders. Several factors may contribute to the development of insomnia in ALS including the features of motor neuron disease (immobilization, cramps and difficulty in turning in bed), disease-related psychological distress and medications (Dauvilliers, 2007; Lo Coco et al., 2011; Panda et al., 2018; Boentert, 2019). Objective features of disrupted sleep quality have also been documented in polysomnographic studies (PSG). PSG studies in ALS documented impaired sleep continuity and alterations of sleep macrostructure (increased N1 sleep, awakenings, wake after sleep onset and arousal index and reduced slow wave sleep, REM sleep and sleep efficiency) (Atalaia et al., 2007; Lo Coco et al., 2011, 2017; Puligheddu et al., 2016; Congiu et al., 2019; Malekshahi et al., 2019). Few studies characterized specific sleep disorders in ALS. The prevalence of restless legs syndrome (RLS) in ALS has been assessed with questionnaires and ranges from 15 to 25% (compared to 1–8% in healthy controls) (Limousin et al., 2011; Lo Coco and La Bella, 2012; Liu et al., 2018; Sun et al., 2021).

Conversely, a handful of studies used PSG to identify other movement disorders in sleep, namely Periodic Limb Movement Disorder and REM sleep behavior disorder (RBD).

Moszczynski et al. (2012) showed that patients with ALS have higher periodic limb movement index (PLMS) than controls (23.55/h vs. 3.28/h), with 54% of patients presenting PLMS > 5/h. Moreover, mortality was higher in ALS patients with PLMS > 5/h than in patients with PLMS < 5/h. By contrast, Puligheddu et al. (2016) found no difference between ALS patients and controls in different PLMS parameters.

Only a few cases of REM sleep behavior disorder have been reported in ALS. Lo Coco et al. (2017) showed that 4.9% of patients with ALS have RBD and REM sleep without atonia. In the study by Puligheddu et al. none of ALS patients had a clinical diagnosis of RBD but REM atonia index was significantly decreased compared to controls and associated with ALSFRS (i.e., REM atonia was higher in ALS patients with more preserved function) (Puligheddu et al., 2016). Finally, circadian sleep/wake rhythm alterations have received little attention (Huang et al., 2018; Dedeene et al., 2019). Only the study by Malekshahi et al. (2019) assessed circadian rhythms in ALS patients by investigating variations of EEG activity. The authors demonstrated the presence of circadian variation in EEG in ALS patients in completely locked-in state suggesting a preserved circadian sleep-wake pattern (Malekshahi et al., 2019).

## Hypothalamic dysfunction and its involvement in ALS-related sleep disorders

Converging evidence has shown a hypothalamic involvement in ALS. Indeed, both histopathologic and volume changes in the hypothalamus of patients with ASL has been documented (Liu et al., 2022). Hypothalamic atrophy affects both the anterior and posterior regions (Liu et al., 2022) and has been documented in both sporadic and familial ALS cases as well as in pre-symptomatic mutation carriers (Gorges et al., 2017). Furthermore, hypothalamic atrophy is not associated with whole-brain atrophy which indicates a region-selective degeneration in ALS. In addition to volumetric changes the hypothalamus of ALS patients also displays phosphorylated 43-kDa TAR DNA-binding protein (pTDP-43) aggregates.

Brettschneider et al. (2013) found pTDP-43 aggregates in 33% to 100% of ALS cases, especially in the lateral hypothalamic region. Similarly, Gabery et al. (2021) found TDP-43 inclusions in the hypothalamus of all ALS patients examined.

The hyperexpression of TDP-43 was also confirmed in mThy1-hTDP-43 transgenic mouse (Scherz et al., 2018). Phosphorylated TDP-43 is closely related to BMI, reflecting its critical role in metabolism, feeding, autonomic, sleep-wake cycle and behavior regulation (Cykowski et al., 2014).

A large amount of evidence on hypothalamic dysfunctions in ALS arises from studies on the GH/IGF-1 system (Ozdinler and Macklis, 2006; Chung et al., 2015).

The GH/IGF-1 system includes GH, Insulin-like growth factor-I (IGF-I) and -II which play a key role in brain growth, development, and metabolism (Gasperi and Castellano, 2010).

Both *in vivo* and animal studies demonstrated GH/IGF system alterations in ALS which may reflect glutamate-induced excitotoxicity (Pellecchia et al., 2010; Chung et al., 2015).

GH deficiency has been documented in a significant portion of ALS patients and resulted predominantly associated with upper motor neuron sign (Morselli et al., 2006; Steyn et al., 2013). Cerebrospinal fluid insulin and IGF-I are also significantly lower in ALS patients compared to controls (Bilic et al., 2006). Conversely, serum IGF-I has been shown to be slightly higher in ALS cases than in controls and very high values IGF-I were associated with a better prognosis (Nagel et al., 2020). More recently, Chung et al. (2015) showed that SOD1G93A transgenic mice exhibit a different pattern of GH secretion during the disease with unchanged GH levels before the onset, higher levels at onset, and lower levels in the late-stage of the disease suggesting that GH can have a protective effect in mutant SOD-1-expressing motor neuronal death.

Other evidence of hypothalamic involvement in ALS comes from clinical trials and mice models on melanocortin (Gasperi and Castellano, 2010; Vercruysse et al., 2016). Indeed, dysfunction of the melanocortin system may lead to peripheral (glucose intolerance) and central alterations (autonomic impairment) (Morselli et al., 2006; Vercruysse et al., 2016).

The hypothalamic-pituitary-adrenal (HPA) axis is another critical system which has been found to be dysfunctional in ALS. Patients with ALS show altered levels of cortisol, which indicates a dysregulation of circadian rhythm of cortisol secretion (Monachelli et al., 2011; Spataro et al., 2015). Cortisol levels are higher in patients than in controls, especially in the morning, in newly diagnosed, spinal-onset and rapid or intermediate progressive phenotypes (Monachelli et al., 2011; Spataro et al., 2015). Some studies hypothesized that the steroid levels could represent a marker of prognosis or susceptibility, as female patients with ALS also show higher levels of testosterone, that did not decrease with age, as in healthy controls (Gargiulo-Monachelli et al., 2014). Steroid levels are positively correlated with respiratory dysfunction in ALS (Gargiulo-Monachelli et al., 2014). In addition, the HPA axis dysfunction is also reflected by the increased levels of progesterone, which correlated negatively with age and positively with survival, time to diagnosis, spinal onset and slow disease progression (Monachelli et al., 2011). A complex interplay between sleep mechanisms and the hypothalamus is increasingly recognized as it is the relation between several features of sleep and the HPA (Lo Martire et al., 2020; Nollet et al., 2020). This association was first reported in Weitzman et al. (1983) who demonstrated that slow wave sleep has an inhibitory effect on the HPA axis and cortisol secretion.

**FIGURE 1:**
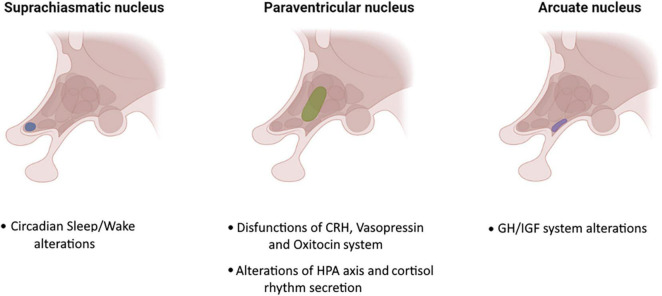
Main hypothalamus nuclei involved in ALS.

Other studies have shown that insomnia, a frequent sleep disorder in ALS, may be related to increased levels of adrenocorticotropic hormone (Vgontzas and Chrousos, 2002). Recently, a growing body of scientific studies have documented alterations in circadian rhythm of cortisol secretion controlled by the HPA axis (Csernansky et al., 2006; Ouanes and Popp, 2019). Higher levels of morning cortisol as well as a higher levels of CSF cortisol have been found in AD and MCI (Csernansky et al., 2006; Popp et al., 2009). Elevated levels of cortisol have been also associated with more rapid cognitive decline in MCI due to AD (Ouanes and Popp, 2019). Furthermore, MCI patients with insomnia had higher cortisol levels and it has been suggested that improving insomnia and consequently cortisol levels may delay further cognitive decline in MCI (Basta et al., 2022). Circadian rhythm dysfunction may also contribute to the progression of neurodegenerative diseases through the modification of molecular, cellular, or physiologic functions (Lauretti et al., 2017). Unfortunately, only few studies investigated the association between pinealocytes/suprachiasmatic neuron loss and sleep alterations in ALS (Huang et al., 2018; Zhang et al., 2018; Dedeene et al., 2019). Huang et al. (2018) performed behavioral and physiological tests on SOD1G93A ALS model mice with and without artificially induced circadian rhythm dysfunction. Mice with circadian dysfunction had earlier symptoms onset, more rapid weight loss and shorter lifespan than mice without circadian dysfunction. Dedeene et al. (2019) performed histological examination on circadian sleep/wake-associated cells (pineal gland and suprachiasmatic nucleus-related hypothalamic neurons) in patients with ALS and/or FTLD-TDP with and without the C9orf72 repeat expansion assessing presence of ALS- and FTLD-TDP-related pathological protein inclusions (DPRs and phosphorylated TDP-43). The authors found an abundance of DPR pathology in the pineal gland of all C9orf72 cases and Poly (GA) inclusions were also identified in SCN-related neurons of C9orf72 cases (Dedeene et al., 2019). Zhang et al. (2018) introduced a point mutation equivalent to the human pathogenic mutation R521C in the rat endogenous *Fus* gene using CRISPR/Cas9 genome editing. The authors showed that pathogenic mutation R521C in *Fus* is associated with sleep-wake alterations and that sleep/wake and circadian abnormalities were the first symptoms presented by gene knock-in mice. Finally, Gabery et al. (2021) documented a selective loss of hypothalamic oxytocin- and orexin-producing neurons in ALS and showed that hypothalamic atrophy and loss of orexin neurons was associated with sleep and eating behavior alterations. A graphical representation of hypothalamic involvement in ALS is shown in Figure 1.

## Discussion

Our analysis of literature data confirms that sleep disorders are common in ALS and have a relevant impact on the prognosis and quality of life of patients. Moreover, a growing body of scientific literature have consistently documented hypothalamic dysfunction in ALS.

Although the number of studies is too limited to draw definitive conclusions, based on the evidence reviewed it is reasonable to hypothesize that hypothalamic dysfunctions may play a key role in the sleep disturbances exhibited by ALS patients.

Sleep abnormalities and circadian rhythm disruption are common in neurodegenerative diseases, (Musiek and Holtzman, 2016; Brzecka et al., 2018; Fifel and Videnovic, 2020). Notably in certain cases sleep and circadian rhythm alterations are considered risk factors for the development of neurodegenerative disorders and may also represent a prodromal marker of neurodegeneration (Musiek and Holtzman, 2016; Tekriwal et al., 2017). However, while sleep and circadian rhythm disruption have been extensively studied in Alzheimer’s, Parkinson’s and Huntington’s disease few studies are available in ALS (Videnovic et al., 2014). The genesis of sleep disorders in ALS seems to be particularly complex, as some sleep disturbances might be secondary to disease-related features while others might be ascribable to hypothalamic dysfunction. The hypothalamus with its nuclei and its connections represents a high-level region of integration of sensory and motor inputs and outputs and it is responsible for the maintenance of energetic homeostasis (Timper and Brüning, 2017). The hypothalamic suprachiasmatic nucleus (SCN) contains the master circadian pacemaker in mammals commonly considered to be the main source of Process C (Stephan and Zucker, 1972; Mouret et al., 1978). Thus, the hypothalamus, regulating all the endogenous rhythms is the controller of the cyclic alternation between sleep and wake and contributes to the alternation between different stages of sleep (NREM and REM sleep) (Stephan and Zucker, 1972; Hiller and Ishii, 2018). Beyond this system, dysfunction of other hypothalamic systems, such as orexin should also be considered (Chieffi et al., 2017; Gabery et al., 2021). Orexin neurons receive input signals from regions related to sleep-wake states, motivation, and visceral cues and in turn, send output signals to a variety of brain regions involved in maintaining wakefulness, regulating REM and NREM as well as to regions involved in responses to rewards, cognition, learning, locomotion and autonomic/sympathetic tone (Chieffi et al., 2017). The dysfunction of the SCN and of the orexin system may be responsible for sleep disorders in ALS including the alteration of the sleep-wake states, disruption of the macrostructure of sleep and EDS (Chieffi et al., 2017).

Interestingly, a possible involvement of orexin in respiratory regulation has also been suggested (Kuwaki, 2010). Studies on orexin-deficient mice have shown frequent sleep apneas and loss of repetitive intermittent hypoxia-induced ventilatory and phrenic long-term facilitation (Kuwaki, 2010). Furthermore, it has been shown that other hypothalamic nuclei are actively involved in respiratory control which are interconnected with respiratory nuclei located in the midbrain, pons, medulla and spinal cord (Fukushi et al., 2019). Even though sleep-related breathing disorders in ALS mainly result from muscle weakness in the pharyngeal muscles, diaphragm, external intercostal and accessory respiratory muscles, the role of the hypothalamus needs to be further investigated. Finally, a series of studies suggested that inflammation and particularly neuroinflammation may be involved in the pathogenesis of sleep disorders in ALS (Liu and Wang, 2017). Altered levels of pro-inflammatory interleukins IL-6 and IL-1β and tumor necrosis factor (TNF) have been described in ALS cases (Hu et al., 2017; Tortelli et al., 2020). Notably, IL-6 and TNF are key molecules in the interactions between sleep and neuroinflammation and have been found to be elevated also in several sleep disorders (Vgontzas and Chrousos, 2002). The complex interplay between neuroinflammation, hypothalamic dysfunction, and sleep disturbances in ALS is intriguing and further research, including preclinical and clinical studies, is needed to gain deeper insights into the underlying mechanisms.

In conclusion, several studies suggest that the hypothalamus represents a pivotal area of interest in the pathogenesis of ALS. Understanding its role in sleep disturbances in ALS may be crucial for the development of new therapeutic opportunities and strategies aimed at improving sleep quality and the quality of life of patients with ALS.

## Author contributions

VG, SZ, and GL: conceptualization. VG, SZ, AG, DU, and GL: methodology. VG, SZ, AG, LT, and MF: data curation. VG, SZ, AG, and DU: writing–original draft preparation. VG, SZ, AG, DU, LT, MF, and GL: writing—review and editing. All authors have read and agreed to the published version of the manuscript.
